# The Pathology of Herpes Zoster

**Published:** 1908-10-10

**Authors:** 


### Pathology.

### THE PATHOLOGY OF HERPES ZOSTER.

It seldom happens that a patient dies when the eruption of shingles is still present, so that it is not often that an opportunity presents itself for investigating the actual lesions in the nervous system that are associated with the disease. The following is a typical case, exhibiting a maximum of inflammatory change in the posterior root ganglion, spreading thence centrifugally for a considerable distance into the posterior nerve root and nerve, and to a less extent centripetally into the posterior horn of grey matter and its connections.

The patient was a woman aged sixty-eight, who came into hospital suffering from zona cervicalis, which had first developed a fortnight previously. The eruption was typical. It extended as high as the lower jaw, and as low as the upper part of the thorax, occupying nearly the whole of the left side of the neck. Five days after her admission the patient developed acute lobar pneumonia, from which she died in a few days. Histological examinations of the cervical nerves and their connections showed the following changes:—

1. In the posterior root ganglion of the third cervical nerve there was active hyperæmia of the minute arterioles and capillaries, with small celled exudation around them; dilatation of the lymphatic spaces, particularly those around the ganglion cells; excess of leucocytes and small round cells in the distended spaces; and loss of protoplasmic definition and nuclear staining-power in the ganglionic cells themselves.

2. In the posterior root ganglion of the second cervical nerve the changes were very much less in degree, but similar in kind, to those in the third ganglion.

3. In the posterior root of the third cervical nerve, both distal and proximal to the ganglion, but particularly the former, there were very acute changes, of which three degrees could be distinguished—namely: (1) in some of the nerve fibres the myelin sheath was still present, but it was broken up at short intervals into balls and fragments; the protoplasm and its nuclei were proliferated; the axis cylinders were indistinguishable, though some of them may have been present still; (2) in the greater number of fibres the proliferation of the nuclei had gone on to such an extent that only a few fragments of myelin could be seen, and the axis cylinders were gone; (3) in a few cases the sheaths of the nerve fibres were quite empty; this may have been due to their contents having fallen out in the process of making the preparations. In the entire section only one or two healthy nerve fibres were still to be detected.

4. In the posterior roots of the second cervical nerve the changes were very much less extensive than they were in the third. Most of the nerve fibres were healthy, but in one or two there was fragmentation of the myelin sheath.

5. In the branches of the cervical plexus there were a great number of perfectly healthy nerve fibres, many of which were doubtless those coming from the anterior roots, and many others those from the comparatively healthy posterior root of the second cervical nerve. Here and there, however, even as far out as the plexus, some fibres exhibited fragmentation of their myelin sheaths and proliferation of the nuclei. Presumably these affected fibres were connected with the posterior root of the third cervical nerve.

6. Sections of the spinal cord at the level of the third cervical segment exhibited much less change than did preparations of the posterior nerve roots themselves. There was, however, distinct hyperæmia of the small arterioles and capillaries in the grey matter of the posterior horn close to the surface, together with distension of the lymphatic spaces, and small round-celled exudation. The nerve cells of the posterior horn stained less deeply on the left side than they did on the right. It is noteworthy that, in this case at any rate, there was no obvious extension of the degenerative process from the nerves of the posterior root into their continuations in the posterior columns of the cord.

The above lesions explain very clearly why the eruption is associated with much pain in many cases; and also why there may be temporary or permanent anæsthesia or paræsthesia after the eruption has disappeared. Irritation of posterior nerve roots causes great pain; a good example of this is seen in cases where the roots are interfered with by secondary deposits of new growth in the vertebræ, or where they are similarly affected by the products of spinal caries. The lightning pains and the various crises of locomotor ataxy are due, in a similar way, to lesions in the posterior nerve roots. The spinal cord itself may be extensively diseased without there being any pain at all unless the posterior nerve roots are affected at the same time.

The pains of herpes zoster are due, in the first instance, to the active hyperæmia and other inflammatory changes in the posterior root ganglion and nerve. If the disease resolves entirely, pain disappears too; if, on the other hand, a residual fibrosis remains, it may persist for months or years after the eruption has gone. This persistence is quite uncommon in children and young adults; but in older persons the reverse is true, and post-herpetic pain may be very troublesome. Perhaps this is due to the tendency for acute inflammations to leave behind them more or less fibrosis in older persons. For pain to remain it is necessary that the posterior nerve fibres should still conduct impulses; in other words, the lesion must stop short of destroying the ganglion cells and their nerve fibres completely. If, on the other hand, the acute disease leads to the complete atrophy of the cells in a posterior root ganglion, instead of pain there will be paræsthesia in the corresponding skin area. As a rule this is not a troublesome symptom.

